# Clinical decision making for using electro-physical agents by physiotherapists, an Israeli survey

**DOI:** 10.1186/s13584-015-0015-x

**Published:** 2015-06-15

**Authors:** Shmuel Springer, Yocheved Laufer, Michal Elboim-Gabyzon

**Affiliations:** Physical Therapy Department, Faculty of Health Sciences, Ariel University, Ariel, 40700 Israel; Physical Therapy Department, Faculty of Social Welfare and Health Sciences, University of Haifa, Haifa, Israel

**Keywords:** Electro-physical agents, Decision-making, Physiotherapy

## Abstract

**Background:**

Electro-physical agents (EPAs) are fundamental components in the management arsenal of physiotherapy. The objective of this study was to provide a comprehensive understanding of the factors affecting the decisions made by Physiotherapists (PTs) when choosing to apply EPAs as a treatment modality.

**Methods:**

A purpose-designed questionnaire was developed to investigate the contribution of 13 factors on the decision to use EPAs. Two hundred questionnaires were randomly distributed to PTs attending the annual conference of the Israeli Physiotherapy Society, 2014. The factors were grouped into six categories and Wilcoxon Sign Rank tests were applied to compare their impact on decision making.

**Results:**

In total, 144 (72%) questionnaires were completed. Good internal consistency was found for the 13 component of the decisions factors (Cronbach’s coefficient alpha = 0.77) with unequal distribution of answers in each question (p < 0.01).

Eighty-one percent of the participants reported past experience, and 55 % mentioned research evidence as strong or very strong factors which influence their decision to use of EPAs. However, only 38% of the participants reported patients’ preferences as a strong or very strong factor. Comparisons between the six categories of the decision factors determined three levels of impact (rank scores) which were significantly different from each other (p < 0.01). Availability of equipment ranked the highest. The lowest level of impact included two categories, technology related issues and patients’ and physicians’ preferences.

**Conclusion:**

The participating PTs were likely to make decisions which were strongly impacted by availability of equipment and operational factors. This research can be used to provide practicing PTs with a basis for a critical appraisal of their decision making regarding the application of EAPs. In addition, due to the strong impact of availability of equipment, health policy makers should verify that the available equipment is up to date with the best research evidence.

## Background

Electro-physical agents (EPAs) are fundamental components in the management arsenal of physiotherapists (PTs), used primarily as adjunct modalities with other forms of treatment. Utilizing different energy forms, EPAs are applied for diagnostic, therapeutic, and feedback purposes [1,2]. However, choosing the beneficial EPA and the most appropriate technique and dosage, are often not simple clinical decisions, bearing in mind that when used inappropriately, EPAs may not only be ineffective, but may have a detrimental effect on a patient’s wellbeing [3].

There is growing research interest in the clinical decision-making skills of health professionals in general, including PTs [4-10]. Addressing decision-making in regard to EPAs, Watson [11] proposed a comprehensive model that includes: identification of the patient’s problems, leading to the establishment of therapeutic goals and priorities; determination of the physiological mechanisms which need to be activated or enhanced; and then selecting the treatment method and dosage which is expected to activate these mechanisms based on the best available evidence. Other factors, in addition to research evidence, that may influence the choice of which EPAs to use include clinical expertise, patient preferences [5,7], and availability of equipment [12].

Several papers have described the frequency of EPAs use and the availability of equipment [13-16]. However, most reports did not address the factors affecting the PTs decision as to when and how to use the EPAs. In an early study regarding the frequency and availability of EPAs, Robinson and Snyder-Mackler [12] also purported to identify factors that affect how and when EPAs are used. However, they only evaluated the availability of equipment and the adequacy of education in clinical electrotherapy, indicating that both these factors significantly affect the frequency of EPA application. Lindsay et al. [17], addressing PTs working at private clinics, reported that the most common reasons for choosing specific modalities are effectiveness, familiarity, ease of application, cost, and safety. However, as this was an early study, it did not consider scientific evidence, referred only to the subjective experience of the therapist, and only provided descriptive data without statistical analysis of the significance of each factor. More recently, Houghton et al. [3] published a comprehensive guideline intended to provide a resource for clinical decision-making regarding EPAs use. The authors indicated that their motivation for developing this guideline was the discrepancies between EPAs educators and course participants in terms of what was considered safe practice with respect to EPAs. Therefore, the focus of their guideline is restricted to contraindications and precautions and does not address other critical components of decision-making.

In light of the limited research on the factors that contribute to decision-making regarding the application of EPAs, the primary objective of this study was to provide a more comprehensive understanding of the factors affecting the decision to use an EPA. A better understanding of the relative contribution of the factors playing a role in these decisions might assist in determining the measures required to ensure the most appropriate application of EPAs.

## Methods

### Procedure

The study was approved by the Institutional Review Board of the Faculty of Social Welfare and Health Sciences at the University of Haifa, Haifa, Israel. A draft questionnaire was developed by the researchers on the basis of previous studies regarding the availability and frequency of EPAs use and factors affecting the decision to use EPAs in practice [12-16]. Content validity of the questionnaire was examined through a rigorous, iterative process with physical therapy faculty involved in EPA education, and with experienced practitioners. This was followed by a pilot test with five PT clinicians who were requested to comment whether the questionnaire items were clear and concise.

The final questionnaire included three main sections. The first section included demographic characteristics of the respondents and questions regarding the availability of equipment and frequency of use. The second section included thirteen factors that may affect the respondent’s decision to use EPAs (decision factors). The participants were requested to note their responses on a five point Likert scale, with 1 indicating no influence on decision and 5 a very strong influence on decision. The last section included questions related to indications for the use of specific electrical stimulation current forms. This is not reported in the present paper.

The survey was conducted during the annual conference of the Israeli Physical Therapy Society, 2014. Two hundred questionnaires were randomly distributed to the PTs attending the conference, who were asked to return the completed questionnaire to the polling booth in the conference hall.

### Data analysis

Descriptive statistics included means, standard deviations, percentages and frequencies, as appropriate. Cronbach’s coefficient alpha test was used to determine the internal consistency of the 13 decision factors. Chi-Square tests for equal proportions were used to examine the differences in the distribution of answers in the decision factors section. Bonferroni corrections were applied to account for multiple comparisons. Consensus was reached by the researchers, who are all expert PT clinicians (each with over 15 years of clinical experience) and educators in the field of EPA, as to the grouping of the decision factors into the following six categories, as follows: (1) Background and experience (items: entry level studies, continuing education, previous clinical experience with EPA and demonstrations of new equipment by medical marketers), (2) Research evidence of efficacy, (3) Technology related issues (items: technophobia, fear of adverse events). (4) Availability of equipment, (5) Operational issues (items: time and ease of application, degree of self-confidence in operating the device, and busy, tight schedule at workplace), and (6) Preferences (items: patient preference and physician prescription).The Wilcoxon Sign Rank test with Bonferroni corrections was applied to compare the degree of impact of each category on the decision to use an EPA. Statistical analyses were performed using SAS 9.3 and Excel (Microsoft Corp., Redmond, WA, USA).

## Results

Among the 200 distributed questionnaires, 144 were completed and returned, indicating a response rate of 72%.The respondents’ characteristics are presented in Table 1. Mean age was 38.3 (±9.42) years, with almost equal distribution between females and males. The majority (98%) was clinical practitioners; with most (62.5%) defining their area of practice as orthopedics and sports. Forty percent of the PTs worked from 20 to 30 hours a week and 51.4% worked more than 30 hours a week. Professional seniority averaged 11.4 (±10.1) years. Participation in postgraduate courses regarding EPAs was noted by 43.1% of the subjects.

**Table 1:** **Demographic characteristics of the study sample (n = 144)**

**Characteristic**	**Mean ± SD or Number (%)**
Age, years	38.3 ± 9.4
Gender	
Male	72 (50.0)
Female	71 (49.3)
Not indicated	1 (0.7)
Degree	
Bachelors (entry level)	144 (100)
Advanced masters in PT	24 (16.7)
Masters in another area	15 (10.4)
Professional seniority, years	11.2 ± 9.9
Area of practice	
Orthopedic and sports rehabilitation	90 (62.5)
Neurologic rehabilitation	29 (20.1)
Women’s health	3 (2.1)
Child development	3 (2.1)
Respiratory rehabilitation	5 (3.5)
Geriatric rehabilitation	10 (6.9)
Education	4 (2.8)
Hours worked per week	
0-10 hours	13 (9.0)
21-30 hours	57 (39.6)
31-40 hours	41 (28.5)
>40 hours	33 (22.9)
Postgraduate courses in EPAs	
Yes	62 (43.1)
No	81 (56.2)
Not indicated	1 (0.7)

EPAs- Electro-physical agents.

Availability of 13 EPAs and their degree of usage is reported in Table 2. The most frequently available agents (≥80%) were transcutaneous electrical nerve stimulation (TENS) (96.5%), heat packs (93.0%), ultrasound (US) (92.3%), cold packs/ice (85.8%), interferential current (IFC) (85.1%) and neuromuscular electrical stimulation (80.0%). The only EPA that was reported as available to 50-79% of the respondents was a whirlpool bath (68.6%). The less available EPAs (available to 30-49% of the respondents) were shortwave (43.6%), biofeedback (36.0%) and functional electrical stimulation (FES) (35.3%). The agents that were available to less than 20% of the PTs were laser (16.6%), shockwave (15.4%) and infrared (6.5%).

**Table 2:** **Availability and use of Electro-physical agents (EPA)**

**EPA**	**Availability % (frequency)**			**Frequency of use: % (frequency)**				
	**Yes**	**No**	**Not sure**	**Once a day**	**Once a week**	**Once a month**	**Seldom**	**Not at all**
Biofeedback EMG	36.0 (49)	53.7 (73)	10.3 (14)	5.3 (5)	5.3 (5)	8.5 (8)	12.8 (12)	68.1 (64)
Cold packs/ice	85.8 (121)	9.9 (14)	4.3 (6)	20.2 (26)	20.9 (27)	14.7 (19)	19.4 (25)	24.8 (32)
FES	35.3 (49)	48.9 (68)	15.8 (22)	16.3 (15)	6.5 (6)	8.7 (8)	12.0 (11)	56.5 (52)
Heat packs	93.0 (133)	6.3 (9)	0.7 (1)	51.1 (68)	24.8 (33)	10.5 (14)	6.1 (8)	7.5 (10)
IFC	85.1 (120)	13.5 (19)	1.4 (2)	48.0 (59)	15.4 (19)	6.5 (8)	10.6 (13)	19.5 (24)
Infrared	6.5 (9)	86.2 (119)	7.3 (10)	5.6 (4)	1.4 (1)	0 (0)	12.7 (9)	80.3 (57)
Laser	16.6 (23)	74.8 (104)	8.6 (12)	6.2 (5)	3.7 (3)	2.5 (2)	11.3 (9)	76.3 (61)
NMES	80.0 (112)	14.3 (20)	5.7 (8)	21.8 (27)	15.3 (19)	18.5 (23)	13.7 (17)	30.7 (38)
Shockwave	15.4 (21)	26.5 (104)	8.1 (11)	10.8 (8)	8.1 (6)	2.7 (2)	9.5 (7)	68.9 (51)
Shortwave diathermy	43.6 (61)	52.1 (73)	4.3 (6)	21.9 (23)	11.5 (12)	1.9 (2)	17.1 (18)	47.6 (50)
TENS	96.5 (138)	2.8 (4)	0.7 (1)	47.8 (66)	21.7 (30)	11.6 (16)	7.3 (10)	11.6 (16)
Ultrasound	92.3 (132)	5.6 (8)	2.1 (3)	38.0 (52)	21.9 (30)	4.4 (6)	16.0 (22)	19.7 (27)
Whirlpool bath	68.6 (96)	28.6 (40)	2.8 (4)	26.1 (30)	19.1 (22)	9.6 (11)	13.0 (15)	32.2 (37)

EPAs- Electro-physical agents, TENS- Transcutaneous electrical stimulation, IFC- Interferential current, NMES- Neuromuscular electrical stimulation, FES- Functional electrical stimulation.

The data on the usage of each EPA are presented in Table 2. The most commonly used EPAs (defined as used by at least 80% of the participants) were heat packs, TENS, IFC and US (92.5%, 88.4%, 80.5%, and 80.3%, respectively). In terms of frequency of use, heat packs were used the most on a daily-week base (51.1%), followed by electrical stimulation for sensory stimulation (IFC and TENS at48.0% and 47.8%, respectively).

Good internal consistency was found for the thirteen components of the decision factors (Cronbach’s coefficient alpha = 0.77), Unequal distribution of answers to each question was demonstrated (p < 0.01). The distribution of each factor is reported in Table 3.Wilcoxon Sign Rank comparisons between the six categories of the decision factors determined three levels of impact (rank scores) which were significantly different from each other (p < 0.01) (see Table 3). Availability of equipment (including only one factor) ranked the highest. The second level of impact included Background and experience, Research evidence of efficacy, and Operational issues, with a total of 8 factors. The lowest level of impact included: Technology-related issues and Preferences, with a total of 4 factors.

**Table 3:** **Factors that influence the decision to use EPAs**

**Factors**	**Level of influence: percentage (frequency)**					Rank score*
	**None (1)**	**Some (2)**	**Medium (3)**	**Strong (4)**	**Very strong (5)**	
**1. Background and experience**						2
Entry level (undergraduate) studies & training background	3.6 (5)	8.6 (12)	28.8 (40)	44.6 (62)	14.4 (20)	
Continuing education studies & training	13.2 (18)	11.1 (15)	22.8 (31)	39.7 (54)	13.2 (18)	
Previous clinical experience with EPSs	1.4 (2)	2.9 (4)	15.1 (21)	49.7 (69)	30.9 (43)	
Demonstration and exposure to new equipment by medical marketers	21.6 (30)	27.3 (38)	36 (50)	12.9 (18)	2.2 (3)	
**2. Research evidence for efficacy**	5 (7)	8.5 (12)	31.9 (45)	39.7 (56)	14.9 (21)	2
**3. Technology-related issues**						3
“Technophobia”	61 (86)	17 (24)	14.9 (21)	5 (7)	2.1 (3)	
Fear of adverse events	30.5 (43)	35.5 (50)	18.4 (26)	12.8 (18)	2.8 (4)	
**4. Availability of equipment**	4.3 (6)	5 (7)	25.7 (36)	35 (49)	30 (42)	1
**5. Operation issues**						2
Time and ease of application	12.8 (18 )	18.4 (26)	32.6 (46)	29.8 (42)	6.4 (9)	
Degree of self-confidence operating the device	9.2 (13)	10.6 (15)	17.7 (25)	44.7 (63)	17.8 (25)	
Busy and tight schedule at workplace	14.2 (20)	20.6 (29)	32.6 (46)	22 (31)	10.6 (15)	
**6. Preferences**						3
Patient preference/request	5.7 (8)	23.6 (33)	32.9 (46)	29.3 (41)	8.5 (12)	
Instruction prescribed by referred physician	38.3 (54)	32.6 (46)	20.6 (29)	6.4 (9)	2.1 (3)	

*The results of Chi-Square Test for Equal Proportions for all the factors were p < 0.01.

## Discussion

The findings regarding availability of EPAs and their frequency of use reported by the respondents in the present study are similar to results of previous studies. As in earlier studies, the most commonly available and frequently used agents were US, heat packs, cold packs/ice, and electrical stimulation for sensory neuromodulation [13,14,18,19]. These findings indicate that the clinical utility of these agents appears to be similar between different countries.

The primary aim of this study was to explore how various factors influence PTs decision to use EPAs. Three levels of impact on decision making were identified with availability of equipment ranked as the most influential factor and technology related issues and patient and physician preferences as the least influential. The findings support the growing understanding that clinical decision making is a complex and multidimensional phenomenon [5]. Therefore, raising awareness of factors that influence decision making together with critical reflection upon the nature of this influence may enhance the quality of clinical decision making.

The evolution of clinical decision making in the physiotherapy is parallel to the growth and maturation of the profession [20]. The ‘Evidence Based Practice’ approach, which has been adopted by the physiotherapy profession as the basis for optimizing and maximizing patient care, considers research evidence, clinician expertise, and patient preferences as the key components in clinical decision making [5,7]. Our findings indicate that PTs indeed consider two of these three components as important factors that influence their decision to use EPAs. Past experience was reported by 80.6% of the participants, and 54.6% mentioned research evidence as strong or very strong factors that influence their decision to use EPA. Although 94.3% of the participants considered patient preference at least to some extent, only 37.8% of the participants reported patient preferences as a strong or very strong factor. Previous studies have identified a low level of shared decision-making between patients and PTs [10,21], demonstrating the need to reinforce the patient’s role in the decision-making process. Though in the current study ‘patient preference’ had the lowest level of impact on decision making (ranked 3), it should be noted, however, that not accepting the patient’s preference does not always imply that decision making was not shared. For example, while a patient may request a specific modality on the basis of information obtained through an advertisement, the decision not to apply this specific modality may be still a shared decision following input received from the clinician. Nevertheless, due to the important role of shared decision making, and the unique and important skills it requires, training for shared decision making should be incorporated in the training of PTs. It should be noted that several medical schools, in Israel and around the world, have begun to take steps in this direction [22].

While, the availability of equipment has been acknowledged as an important factor considered when using EPAs [12], the category ‘availability of equipment’ was ranked in the present study as the most influential one. In should be noted, that while the factors were listed in the questionnaire as independent factors, there was no directive indicating the responders to consider each factors separately disregarding availability of equipment, thus, probably increasing the significance of the ‘availability of equipment’ factor. Yet, still the fact that equipment availability was ranked the highest was surprising. Although not directly investigated in this study, it is likely that when the PTs considered availability of EPAs, they did not only refer to whether EPA equipment is present in the clinic, but also to the degree to which it is available for their use when needed. For example, while an US device may be present in the clinic, due to multiple users and a tight schedule (operational issues), the therapist might have considered it as not sufficiently available.

The impact of equipment availability on the decision-making process is concerning. It may indicate that under some circumstances, such as busy schedules at the workplace, PTs may not use EPAs even if there is a clinical rationale for this intervention. It should be noted that a similar trend was reported in a recent study that tested the compliance of PTs with a clinical practice guideline for the treatment of low back pain developed by the National Institute for Clinical Excellence. The PTs strongly believed in the principles of the guideline, and thought them relevant to their practice. Yet, they mentioned that the guideline’s recommendations were not realistic in day-to-day practice and therefore were not always adopted [23].

In the present study, the impact of the ‘background and experience’ category was ranked second after ‘availability of equipment’. Within this category, previous experience was considered more influential than any of the other three factors (entry level education, continuing education and demonstrations of new equipment). Despite the widespread use of EPAs within physiotherapy practice, previous studies indicate that EPAs are often poorly understood [11]. A review by Shah and Farrow [14] intended to describe EPAs usage from 1990 to 2010 suggested that lack of knowledge and training may be a common reason for reduced use of EPA modalities. Furthermore, a recently published study found that new physiotherapy graduates may feel not confident in selecting and using EPAs on entering the workforce [24]. The findings which reflect that previous experience was considered more influential than educational factors, and that only 43.1% of the participants reported having taken a post-graduate course regarding EPAs usage, may encourage clinicians to consider to enhance their knowledge regarding EPAs.

The present study has several limitations. It included a relatively small convenience sample of therapists who attended the Israeli Physical Therapy Society Annual Conference which may have introduced a selection bias, as therapists attending a conference may not necessarily represent all therapists in the population. A recent report by the Israeli Ministry of Health has shown that most of the PTs in Israel are between the ages of 31–44 [25]. It should be noted that the age distribution in the present sample is consistent with this report. As with all surveys there might be additional potential selection bias, as those with strongly negative or positive views may have been more likely to participate. However, this bias seems unlikely as the participants’ responses were varied and presented different approaches towards the consideration for using EPAs. Furthermore, some participants did not answer all questions, and the study did not include separate analyses based on area of practice and seniority .Future studies with larger samples should be conducted to evaluate whether background variables effect clinical decision making regarding use of EPAs. Finally, this study did not refer separately to each modality. It is neither possible nor appropriate to draw conclusions regarding decision making for all EPAs as a group. Thus, additional research that focuses on specific EPAs is suggested.

## Conclusions

Decision making regarding the use of EPAs was found to be multi-dimensional Availability of equipment was ranked by the participants as the most influential category effecting the decision to use EPAs, with the impact of ‘background and experience’, ‘research evidence of efficacy’, and ‘operational issues’ ranked at a somewhat lower level. The study offers a framework for PTs to evaluate their decision making process regarding the application of EPAs, which may enhance the quality of care provided to their patients. In addition, due to the strong impact of availability of equipment, health policy makers should verify that the available equipment is up to date with the best research evidence.

## Abbreviations

EPAs: Electro-physical agents
PTs: Physiotherapists
US: Ultrasound
TENS: Transcutaneous electrical nerve stimulation
IFC: Interferential current
